# Human T-Lymphotropic Virus Type 1 (Htlv-1): a new subgroup within the cosmopolitan subtype

**DOI:** 10.1186/1742-4690-8-S1-A75

**Published:** 2011-06-06

**Authors:** María Eirin, Leandro Jones, Carolina Berini, Darío Dilernia, Cecilia Delfino, Mirna Biglione

**Affiliations:** 1 Centro Nacional de Referencia para el SIDA, Facultad de Medicina, UBA, Buenos Aires, Argentina; 2 División de Biología Molecular, Estación de Fotobiología Playa Unión, Playa Unión, Chubut, Argentina

## Introduction

HTLV-1 is classified in seven subtypes (a-g) most of them restricted to specific regions (b-e), while the Cosmopolitan subtype (a) is worldwide distributed. Cosmopolitan subtype has experimented a degree of molecular diversity giving rise to five subgroups: Transcontinental (A), Japanese (B), West African (C), North African (D) and Black Peruvian (E).

## Objective

to confirm the classification of four HTLV-1 highly divergent strains as a new subgroup within the Cosmopolitan subtype.

## Materials and methods

LTR sequences from 65 HTLV-1 positive Buenos Aires residents were retrospectively studied. Phylogeny of LTR region was studied by three different methods (ML, MP and NJ). A similitude index (SI) was estimated as the mean number of nucleotide substitutions from each subgroup (intra-subgroup) or between sequences from a given subgroup against all from other subgroups (inter-subgroup) (script described in the R Statistical Package Language http://www.R-project.org).

## Results

The three phylogenetic methods were consistent, showing a well supported monophyletic clade that included two Peruvian sequences clustering with two references (Bl3 from Peru and Br4 from Brazil) previously described as divergent, branching off all known subgroups (82% bootstrap, ML). The similitude analysis showed a SI intra-subgroup similar to those obtained for A-D subgroups and a SI inter-subgroup similar to subgroups A and D. Following nomenclature, it was named subgroup F.

## Conclusions

This study confirms the existence of a highly divergent monophyletic clade within the Cosmopolitan subtype composed of sequences from Peruvian and Brazilian individuals and suggest that more studies should be performed in this South-American area where the last subgroups (E and F) has been detected.

